# Safety and efficacy of topical OCS-01 ophthalmic suspension for the treatment of diabetic macular oedema: stage 1 of a multicentre, double-blind, randomised, vehicle-controlled phase 2/3 trial

**DOI:** 10.1016/j.eclinm.2026.104091

**Published:** 2026-07-27

**Authors:** Arshad M. Khanani, Ramin Tadayoni, Sabri Markabi, Sunil Patel, Hani Salehi-Had, David S. Boyer

**Affiliations:** a Sierra Eye Associates and The University of Nevada, Reno School of Medicine, Reno, NV, USA; b Ophthalmology Department, Fondation Adolphe de Rothschild and the Lariboisière and Saint-Louis University Hospitals, Paris, France; c Health R&D, LLC, Miami, FL, USA; d Ophthalmology Specialists of Texas, PLLC, Abilene, TX, USA; e Retina Associates of Southern California, Huntington Beach, CA, USA; f Retina-Vitreous Associates Medical Group, Los Angeles, CA, USA

**Keywords:** Diabetic macular oedema, Topical ocular treatment, OCS-01 ophthalmic suspension, Dexamethasone

## Abstract

**Background:**

Topical ocular treatments for diabetic macular oedema (DMO) have not previously been considered effective. We aimed to assess the efficacy and safety of topical OCS-01 (dexamethasone 1.5% ophthalmic suspension) in the treatment of DMO.

**Methods:**

This is a multicentre, double-blind, randomised, vehicle-controlled phase 2/3 trial. The objective of Stage 1 (reported herein, Stage 2 is ongoing) was to evaluate the efficacy and safety of a dosing regimen of OCS-01 to use in Stage 2, specifically to estimate the magnitude of the treatment effect on BCVA. Stage 1 was conducted at 27 ophthalmology clinics in the USA and four in Hungary. Patients aged 18–85 years with DMO involving the central macula (central subfield thickness (CST) ≥310 μm by SD-OCT), and ETDRS letter score 24–65 in the study eye were included in the study. Key exclusion criteria were macular oedema with causes other than DMO, decreased BCVA due to causes other than DMO, significant macular ischemia which would have prevented gain in BCVA, or other ocular disease that may have caused substantial reduction in BCVA. Patients were randomised, using an automated interactive response system to generate randomisation codes, stratified for baseline BCVA (<60 versus ≥60) and lens status (phakic or nonphakic), in blocks of 6 in a 2:1 ratio to receive topical ocular OCS-01 or matching vehicle. Treatment was given six times/day for 6 weeks (loading phase), followed by three times/day for 6 weeks (maintenance phase). Patients, investigators and the study team were masked to treatment assignment. Masking of treatment was maintained by using a vehicle that was identical to OCS-01 but did not contain dexamethasone, and OCS-01 and vehicle were supplied in identical packaging. The primary endpoint was change from baseline to Week 6 in ETDRS letter score in the Intent-to-Treat Set (all randomised patients). Safety analyses were based on the Safety Set (all patients who received ≥1 dose of study drug). The trial is registered with ClinicalTrials.gov, NCT05066997.

**Findings:**

Between Oct 5, 2021 and March 16, 2023, 100 patients (53 male, 47 female) received OCS-01 and 48 (26 male, 22 female) received vehicle; 85 and 39 patients, respectively, completed the study. LS mean (SE) change from baseline to Week 6 in BCVA ETDRS letter score was significantly greater with OCS-01 than Vehicle: 7.2 (0.85) letters versus 3.1 (1.26) letters (LS mean difference 4.1 (1.52) letters, 95% CI 1.1,7.0, p = 0.0070). Ocular adverse events (AEs) in the study eye occurred in 47/100 (47%) patients in the OCS-01 group and 22/48 (46%) Vehicle patients.

**Interpretation:**

Whilst acknowledging that no formal hypothesis was tested in Stage 1, topical OCS-01 was well tolerated, with no unexpected safety findings, and improved visual acuity in patients with DMO over the 12-week duration of Stage 1 of this study. Further data are required. The dosing regimen was found to be appropriate for use in Stage 2, which will provide longer-term data.

**Funding:**

Oculis Sàrl, Lausanne, Switzerland.

**Research in context**

**Evidence before this study**

We searched PubMed from 1st January 2000 to 6th February 2025, without language restrictions, using the search terms “diabetic macular oedema”, and “clinical trial” and identified 780 publications, of which 570 reported data from clinical trials. Of these, three described topical treatments, the rest intravitreal, laser or systemic therapies: one reported that topical bromfenac and betamethasone had no effect on best corrected visual acuity (BCVA) or central macular thickness (CMT). A second publication reported a non-comparative study using a 1.5% dexamethasone-cyclodextrin eye drop suspension in which statistically significant improvements from baseline in both BCVA and CMT were observed. The third publication described a Phase 2 study with OCS-01 (1.5% dexamethasone) eye drops, in which mean CMT showed a greater decrease from baseline with OCS-01 than vehicle; BCVA also showed greater improvements with OCS-01 than vehicle, although the difference was not statistically significant.

**Added value of this study**

Stage 1 of this randomized, double-blind, vehicle-controlled, multicentre study in patients with DME aimed to confirm the dosing regimen of topical OCS-01 to be used in Stage 2 of the study. The study demonstrated, for the first time in a vehicle-controlled study, statistically significant effects of OCS-01 on both BCVA and macular thickness in patients with DME. OCS-01 was well-tolerated, with no unexpected adverse events.

**Implications of all the available evidence**

Although limited by treatment duration, this study adds to the evidence that topical therapy with OCS-01 for DME could complement existing intravitreal treatments. Stage 2 of this study will provide longer-term evidence that should clarify the potential role of topical therapy in the treatment of DME.

## Introduction

Diabetic macular oedema (DMO) is a common complication of diabetes mellitus, with a global prevalence estimated at 6.8% among patients with diabetes.^1^ Untreated DMO may be associated with decreased visual acuity, particularly with foveal involvement.^2^ The pathophysiology of DMO is complex, but it is thought that a decrease in retinal oxygen tension due to hyperglycaemia upregulates synthesis of vascular endothelial growth factor (VEGF) and other permeability factors, leading to microvasculature leakage.^3^ Current FDA-approved treatments for DMO are intravitreal injections of VEGF inhibitors such as ranibizumab, aflibercept, brolucizumab, and faricimab (also bevacizumab, which has been used off-label for many years)^3^, ^4^, ^5^, ^6^, ^7^, ^8^, ^9^, ^10^, ^11^ or intravitreal corticosteroid implants that release drug over 3–6 months or up to 3 years.^12^, ^13^, ^14^ Corticosteroids decrease VEGF production directly and by reducing inflammation.^3^ Treatment guidelines for central-involved or moderate to severe DMO recommend the use of intravitreal therapies, primarily VEGF inhibitors, but also corticosteroid implants.^15^, ^16^, ^17^

Intravitreal therapies are effective, but involve discomfort for patients and carry risks of complications including subconjunctival haemorrhage, intraocular inflammation, retinal tears, endophthalmitis, and vitreous haemorrhage. Corticosteroid implants are also associated with increased intraocular pressure (IOP) and cataract development. Access to intravitreal therapies may be limited by geography and financial considerations, given that specialised facilities and highly-skilled personnel are required.^3^ Topical DMO therapies could potentially decrease risks, reduce costs, be more tolerable for patients, and improve treatment accessibility. Achieving therapeutic concentrations of topically administered compounds in the posterior segment of the eye has not previously been considered to be practicable, however.^18^

OCS-01, an OPTIREACH® formulation of high-concentration dexamethasone eye drop (Oculis Operations Sàrl, Lausanne, Switzerland), is an eye drop formulation containing 1.5% dexamethasone. OCS-01 was developed to increase drug concentrations in both anterior and posterior segments of the eye following topical application. A formulation technology based on γ-cyclodextrin provides a stable ophthalmic suspension with a higher concentration of active drug than existing commercial dexamethasone ophthalmic suspensions. In animal models, topical administration resulted in therapeutic drug concentrations in both anterior and posterior segments.^19^

A multicentre, randomised, double-masked, parallel-group, vehicle-controlled Phase 2 study (EudraCT 2017–001172-36) in which patients with DMO received OCS-01 or vehicle at a dosage of one drop three times/day for 12 weeks^20^ showed significantly greater improvements in both visual acuity and central macular thickness with OCS-01 as compared to vehicle. Here, we describe Stage 1 of a two-part Phase 2/3 study with OCS-01 in the treatment of DMO (NCT05066997).

## Methods

### Study design and ethics

DIAbetic Macular oedema patients ON a Drop (DIAMOND, DX-219) is a two-stage, multi-centre randomised, double-masked, parallel-group, vehicle-controlled trial. Stage 1 was completed and is reported here. Stage 2 is ongoing and will be reported separately. Patients were enrolled between Oct 5, 2021, and March 16, 2023 at ophthalmology clinics, 27 sites in the USA and four sites in Hungary.

The protocol was approved by independent ethics committees and/or institution review boards for each study centre. Ethics approval numbers are listed within the Supplementary Appendix. The trial was performed in accordance with the principles of the Declaration of Helsinki. The authors assume responsibility for the accuracy and completeness of the data and analyses, and for the fidelity of the trial and this report to the protocol. The study is reported in accordance with the CONSORT guidelines. The study protocol and statistical analysis plan are available online. The study is registered with ClinicalTrials.gov (NCT05066997).

### Participants

Eligible patients could be male or female, 18–85 years of age, with Type 1 or 2 diabetes mellitus. A diagnosis of DMO was required, with best corrected visual acuity (BCVA) Early Treatment Diabetic Retinopathy Study (ETDRS) letter score of 24–65 (Snellen 20/320 to 20/50), with retinal thickening (central subfield thickness [CST] at least 310 μm) in the study eye due to DMO involving the central macula per evaluation by a central reading centre (Merit CRO, Inc, Madison, WI), based upon spectral domain optical coherence tomography (SD-OCT) at screening. Patients were either anti-VEGF and corticosteroid intravitreal treatment-naïve in the study eye or underwent an agreed washout period for these compounds. Complete entry criteria are provided in the Supplementary Appendix. All patients provided written informed consent.

### Randomisation and masking

In Stage 1 of the study, patients were randomised in a 2:1 ratio to receive OCS-01 or matching vehicle. Patients were electronically assigned a randomisation number after qualifying for study entry and randomisation was performed using an interactive response system to generate a randomisation code to assign treatment. Randomisation was stratified for baseline BCVA (<60 letters versus ≥60 letters) and lens status (phakic or nonphakic), in blocks of 6. To maintain masking, OCS-01 and Vehicle were prepared and provided in identical packaging. Randomisation codes were to be broken only in the event of a medical emergency, or when knowing the treatment assignment was absolutely necessary for the medical management of the patient.

### Procedures

Patients received treatment in the study eye, six times/day for 6 weeks (the loading phase), then three times/day for 6 weeks (the maintenance phase). The Vehicle did not contain dexamethasone but was otherwise identical to OCS-01. Patients were assessed for efficacy and safety at baseline and then every 2 weeks. Visual acuity was assessed using standardised methods; each site was certified by an independent organization that checked technicians, facilities and equipment to assure standardization. Central subfield thickness was determined by SD-OCT.

### Outcomes

The primary study endpoint was mean change from baseline to Week 6 in BCVA ETDRS letters score. Secondary endpoints were the proportion of patients with ≥15 letters gain from baseline in BCVA ETDRS letters at Week 6; the area under the curve (AUC) of BCVA ETDRS letters changes from baseline across post-baseline visits up to Weeks 6 and 12; mean change in BCVA ETDRS letters score at Week 12 compared with baseline and Week 6; proportion of patients with a ≥15 letters gain in BCVA ETDRS letters at Week 12 compared with baseline and Week 6; mean change in CST at Weeks 6 and 12 compared with baseline, and at Week 12 compared with Week 6; the proportion of patients with a ≥5 letters or ≥10 letters gain from baseline in BCVA ETDRS letters at each post-baseline visit; proportions of patients with ≥3-, 2- or 1-line gain in BCVA compared to baseline at each post-baseline visit, and mean change from baseline in CST at each post-baseline visit. Safety assessments included reporting of adverse events, slit lamp biomicroscopy, lens clarity grading assessment (using the AREDS Clinical Lens Grading Protocol),^21^ IOP assessment (using a calibrated Goldmann application tonometer affixed to a slit lamp, with the same instrument used at every visit if possible), and dilated indirect ophthalmoscopy.

### Statistical analysis

The main goal of Stage 1 of the study was to confirm the final design of Stage 2, particularly the dosing regimen, by evaluating the safety and efficacy (and specifically to estimate the magnitude of the treatment effect on BCVA) of the chosen OCS-01 dose regimen versus vehicle, and to confirm assumptions made regarding sample size for Stage 2.

While no formal hypothesis was tested in Stage 1, the sample size of 132 study eyes (patients) (88 patients in OCS-01 treatment group, 44 patients in Vehicle treatment group) within Stage 1 was based on a power calculation for a 2-sample t-test. Assuming a difference in the mean change from baseline BCVA ETDRS letters score to Week 6 of 2.8 letters between OCS-01 and Vehicle in the absence of withdrawals, based on previous data,^20^ a common standard deviation in the change from baseline in BCVA ETDRS letters score to Week 6 of 6.0 letters within each treatment group, a 1-sided alpha = 0.05, and a 2:1 randomization, 132 study eyes (patients) are required to achieve a power of 80%. Assuming a 10% withdrawal rate through Week 12, a total of approximately 147 patients were randomised.

All efficacy analyses were based on the Intent-To-Treat (ITT) Set (all patients screened, enrolled, and randomised). Patients were analysed according to randomised treatment. All safety analyses were based on the Safety Set (all patients who were administered at least 1 dose of the study drug); patients were analysed according to treatment received.

Efficacy endpoints were summarised descriptively. For continuous endpoints, the number of patients, mean, standard deviation, median, minimum, and maximum were presented. For categorical endpoints, the numbers and percentages were presented. All efficacy analyses used a 2-sided alpha = 0.05 statistical test and corresponding 2-sided 95% CIs were presented where applicable. For the primary analysis, a mixed model for repeated measures was used. The model included fixed effects for treatment and visit, interaction between treatment and visit, and baseline BCVA ETDRS letter score as a covariate. The least squares (LS) mean and standard error for each treatment group, LS mean treatment difference (OCS-01 – Vehicle), associated 95% CI, and p-values were provided at each visit. The regression coefficient for the treatment variable, its p-value, and 95% CI were presented.

The AUC of change in BCVA ETDRS letter score from baseline to Week 6 was computed using the trapezoidal rule on observed data, and summarised descriptively; AUC was compared between treatment groups using an ANCOVA model with treatment as a factor and baseline BCVA ETDRS letter score as a covariate. LS means and associated 95% CIs for each treatment and the LS mean difference between treatments (OCS-01 minus Vehicle) and associated 95% CI were calculated.

Missing data for efficacy endpoints were imputed based on a pattern-mixture model approach. Intermittent missing (non-monotone) data, or missing data without withdrawal, were first imputed using Markov Chain Monte Carlo to obtain a monotone missing pattern. Then post-withdrawal missing data (monotone) were multiply imputed separately for patients in the OCS-01 group and in the Vehicle group. Missing data with withdrawal for AEs or lack of efficacy were assumed to be missing not at random (MNAR) after withdrawal date. Missing data without withdrawal or with withdrawal for reasons other than AEs or lack of efficacy were assumed to be missing at random (MAR). For patients in the Vehicle group (MAR or MNAR) and patients in the OCS-01 group with MNAR data, missing data were multiple imputed using Vehicle-based regression methodology. For patients in the OCS-01 group with MAR data, missing data were multiple imputed using treatment-based regression methodology. Three sensitivity analyses were performed for the primary endpoint, assuming all missing data to be MAR, all to be MNAR, or using observed data only with no imputation of missing values.

All analyses were performed using SAS Version 9.4.

### Role of the funding source

The sponsor was involved in the study design; the collection, analysis and interpretation of data; and the writing of the report. The authors had full access to the data in the study, and all authors agreed to submit the manuscript for publication.

## Results

### Patients

**Fig. 1:**
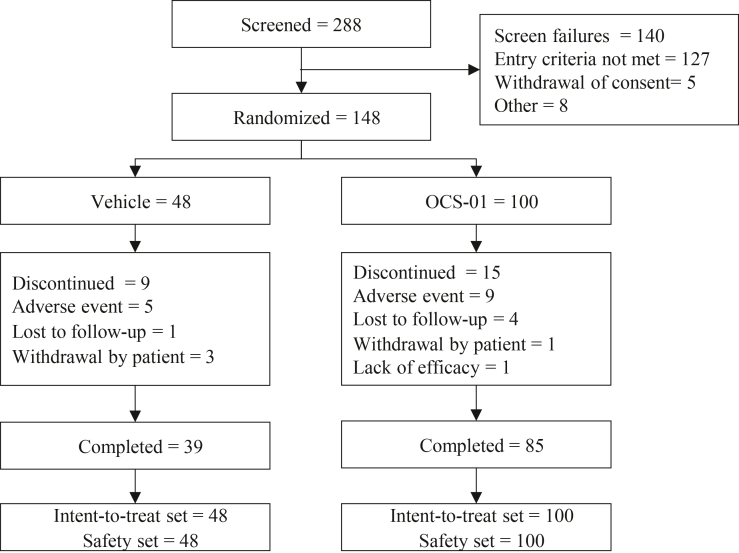
Patient disposition (ITT set).

Patients were recruited between Oct 5, 2021 and March 16, 2023. 288 patients were screened and 148 were randomised, 48 to vehicle and 100 to OCS-01 (Fig. 1). The majority of patients (124/148, 84%) completed the study; the most common reasons for discontinuation were adverse events (14/148, 9.5% of patients) and being lost to follow up (5/148, 3.4%). There were no major differences between treatment groups in rates of, or reasons for, discontinuation. Major protocol deviations occurred in 7/100 (7%) of the OCS-01 group (three patients using prohibited concomitant medication and four patients with incorrect entry criteria) and 3/48 (6%) of the vehicle group (one patient using prohibited concomitant medication, one patient received incorrect study medication and one patient had a randomisation error). All randomised patients were included in the ITT and Safety Sets.

Treatment groups were generally well-matched for demographic and baseline disease characteristics (Table 1). Mean (±SD) age was 62.6 ± 8.5 years, 69/148 (47%) of patients were female, and 116/148 (78%) of patients were Caucasian, 18/148 (12%) were Black/African American, and 6/148 (4%) were Asian. The study eye was the left eye in 88/148 (60%) of patients, 108/148 (73%) of eyes were phakic, and the most common iris colour was brown (93/148, 63%). Mean (±SD) time from diagnosis of DMO to baseline was 2.0 ± 2.6 years, mean (±SD) BCVA was 57.7 ± 8.72 letters and mean (±SD) CST was 450.5 ± 125.5 μm. Median duration of DMO was higher in the OCS-01 group (0.76 years) than in the Vehicle group (0.26 years), but mean and median BCVA and CST were very similar between groups. Prior anti-VEGF treatment was also similar between groups, with bevacizumab the most commonly used therapy (in 35/148, 24% of patients), followed by aflibercept (in 23/148, 16% of patients).

**Table 1:** Baseline demographic and disease characteristics (ITT set).

	OCS-01 N = 100	Vehicle N = 48	Total N = 148
Age, years			
Mean (SD)	61.9 (9.01)	63.9 (7.34)	62.6 (8.53)
Sex, n (%)			
Male	53 (53.0)	26 (54.2)	79 (53.4)
Female	47 (47.0)	22 (45.8)	69 (46.6)
Ethnicity, n (%)			
Not Hispanic or Latino	68 (68.0)	34 (70.8)	102 (68.9)
Hispanic or Latino	32 (32.0)	13 (27.1)	45 (30.4)
Not reported/unknown	0	1 (2.1)	1 (0.7)
Race, n (%)			
White	81 (81.0)	35 (72.9)	116 (78.4)
Black or African American	12 (12.0)	6 (12.5)	18 (12.2)
Asian	4 (4.0)	2 (4.2)	6 (4.1)
American Indian or Alaska Native	1 (1.0)	2 (4.2)	3 (2.0)
Other	1 (1.0)	2 (4.2)	3 (2.0)
Not reported/unknown	1 (1.0)	1 (2.1)	2 (1.4)
Study eye, n (%)			
Left	58 (58.0)	30 (62.5)	88 (59.5)
Right	42 (42.0)	18 (37.5)	60 (40.5)
Lens status			
Phakic	71 (71.0)	37 (77.1)	108 (73.0)
Non-phakic	29 (29.0)	11 (22.9)	40 (27.0)
Iris colour, n (%)			
Brown	63 (63.0)	30 (62.5)	93 (62.8)
Blue	24 (24.0)	8 (16.7)	32 (21.6)
Green	8 (8.0)	4 (8.3)	12 (8.1)
Hazel	4 (4.0)	6 (12.5)	10 (6.8)
Gray	1 (1.0)	0	1 (0.7)
Duration of DME diagnosis (years)^a^			
Mean (SD)	2.004 (2.6149)	1.932 (2.6560)	1.981 (2.6192)
Median (min, max)	0.756 (0.04, 14.00)	0.263 (0.04, 8.84)	0.522 (0.04, 14.00)
BCVA ETDRS letter score			
Mean (SD)	57.5 (9.26)	58.3 (7.51)	57.7 (8.72)
Median (min, max)	61.0 (25, 65)	62.0 (33, 65)	61.0 (25, 65)
CST (μm)			
Mean (SD)	452.95 (131.805)	445.29 (112.459)	450.47 (125.525)
Median (min, max)	406.50 (264.0, 770.0)	425.00 (293.0, 803.0)	408.50 (264.0, 803.0)
Prior anti-VEGF treatment, n (%)			
Bevacizumab	21 (21.0)	14 (29.2)	35 (23.6)
Aflibercept	16 (16.0)	7 (14.6)	23 (15.5)
Ranibizumab	3 (3.0)	2 (4.2)	5 (3.4)
Brolucizumab	1 (1.0)	0	1 (0.7)

BCVA, best corrected visual acuity; CST, central subfield thickness; DME, diabetic macular oedema; ETDRS, Early Treatment Diabetic Retinopathy Study; ITT, intent-to-treat.

a The length of time since DME diagnosis in the study eye was derived using DME start date in the study eye to baseline date; these data were available for 47 patients in the Vehicle group and 99 patients in the OCS-01 group. Sex and race were self-reported by patients.

Median exposure to study drug was 85.0 days in both treatment groups, range 1–92 days in the Vehicle group and 13–128 days in the OCS-01 group.

### Primary efficacy outcome

Using the mixed model for repeated measures, the LS mean (SE) change from baseline to Week 6 (end of loading phase) in BCVA ETDRS letter score was 7.2 (0.85) letters in the OCS-01 group and 3.1 (1.26) letters in the Vehicle group, with an LS mean difference 4.1 (1.52) letters, 95% CI 1.1, 7.0, p = 0.007 (Table 2). The three sensitivity analyses for the primary endpoint, assessing the impact of missing data showed similar results to the primary endpoint (Supplementary Table S1 in the Supplementary Appendix). Supplementary Fig. S1 in the Supplementary Appendix shows BCVA at each visit up to Week 12.

**Table 2:** Primary efficacy analysis: change in BCVA ETDRS letter score from baseline to Week 6.

Visit	Statistics	OCS-01 (N = 100)		Vehicle (N = 48)	
		Actual value	Change from baseline	Actual value	Change from baseline
Baseline	Mean (SD)	57.5 (9.26)	7.4 (9.01)^b^	58.3 (7.51)	3.2 (8.73)^b^
Week 6	Mixed model for repeated measures^a^				
	LS mean (SE)		7.2 (0.85)		3.1 (1.26)
	95% CI		(5.5, 8.8)		(0.6, 5.6)
	OCS-01 - Vehicle LS mean difference (SE)^c^	4.1 (1.52)			
	95% CI	(1.1, 7.0)			
	p-value	0.007			

BCVA, best corrected visual acuity; ETDRS, Early Treatment Diabetic Retinopathy Study; ITT, intent-to-treat; LS, least squares.

Note: Baseline was defined as the last non-missing measure prior to initiation of study drug.

a The change from baseline compared between treatment groups was analysed with a mixed model for repeated measures using an Autoregressive 1 covariance structure, patient as a random effect, fixed effects for treatment and visit, the interaction between treatment and visit, and baseline BCVA ETDRS letter score as a covariate. Imputation rules were applied based on a pattern-mixture model approach. Table header N’s indicate the number of patients contributing data to the statistical modelling.

b No statistical testing was performed on the unadjusted mean change from baseline.

c LS mean difference OCS-01–Vehicle.

### Secondary efficacy outcomes

The proportions of patients with a ≥15 letters (Table 3), ≥10 letters (Supplementary Table S2 in the Supplementary Appendix) or ≥5 letters (Supplementary Table S3 in the Supplementary Appendix) gain from baseline at Weeks 6 and 12 were higher for OCS-01 than vehicle. Supplementary Table S4 in the Supplementary Appendix shows other secondary efficacy outcomes based on BCVA letters score. The mean change from baseline to Week 12 in BCVA ETDRS letters score was also higher in the OCS-01 group than the vehicle group, as was the AUC of BCVA ETDRS letters changes from baseline across post-baseline visits up to Weeks 6 and 12. Only a small proportion of patients in each group showed a 3-line (15 letters) or greater gain from Week 6 to Week 12 (Table 3; Supplementary Table S5 in the Supplementary Appendix shows proportions of patients in each group with a 3-line or greater gain from baseline at each visit). The OCS-01 group also showed higher proportions of patients with at least 2-line or at least 1-line gains from baseline in BCVA at each post-baseline visit (Supplementary Tables S2 and S3 in the Supplementary Appendix).

**Table 3:** Proportions of patients with a 15-letter or greater gain from baseline in BCVA ETDRS letters at Weeks 6 and 12.

Visit	Statistics	OCS-01 (N = 100)	Vehicle (N = 48)
15-letter or greater gain in BCVA at Week 6 compared with baseline^a^ (ITT set)			
Week 6	N	95	41
	≥15 letters gain (response), n (%)	24 (25.3)	4 (9.8)
	<15 letters gain, n (%)	71 (74.7)	37 (90.2)
	Estimated marginal effects of treatment	24.43	9.34
	95% CI	15.93, 32.93	0.65, 18.03
	Difference in marginal effects (OCS-01 – Vehicle)	15.09	
	95% CI	2.90, 27.29	
	p-value	0.015	
	Adjusted odds ratio (OCS-1/Vehicle)	3.17	
	95% Wald CI for odds ratio	1.02, 9.84	
	p-value	0.046	
15-letter or greater gain in BCVA at Week 12 compared with baseline^a^			
Week 12/EOS	N	84	40
	≥15 letters gain (response), n (%)	23 (27.4)	3 (7.5)
	<15 letters gain, n (%)	61 (72.6)	37 (92.5)
	Estimated marginal effects of treatment	23.76	7.81
	95% CI	15.26, 32.26	−0.46, 16.09
	Difference in marginal effects (OCS-01 – Vehicle)	15.95	
	95% CI	4.00, 27.90	
	p-value	0.009	
	Adjusted odds ratio (OCS-1/Vehicle)	3.75	
	95% Wald CI for odds ratio	1.07, 13.23	
	p-value	0.039	
15-letter or greater gain in BCVA at Week 12 compared with Week 6^b^			
Week 12/EOS	N	84	39
	≥15 letters gain (response), n (%)	3 (3.6)	1 (2.6)
	<15 letters gain, n (%)	81 (96.4)	38 (97.4)
	Estimated marginal effects of treatment	3.37	2.16
	95% CI	−0.33, 7.07	−1.18, 5.50
	Difference in marginal effects (OCS-01 – Vehicle)	1.21	
	95% CI	−3.79, 6.20	
	p-value	0.636	
	Adjusted odds ratio (OCS-1/Vehicle)	1.71	
	95% Wald CI for odds ratio	0.20, 14.80	
	p-value	0.628	

BCVA, best corrected visual acuity; EOS, end of study; ETDRS, Early Treatment Diabetic Retinopathy Study; ITT, intent-to-treat.

The model used was a generalized estimating equation for binary response model with fixed effects for treatment and visit, the interaction between treatment and visit, and baseline BCVA ETDRS letter score as a covariate. Estimated marginal effects, difference in estimated marginal effects, and CI limits from analysis were multiplied by 100.

Imputation rules were applied based on a pattern-mixture model approach. Table header N’s indicate the numbering data to the statistical modelling and n indicates the number of patients with data prior to imputation.

a Baseline was defined as the last non-missing measure prior to initiation of study drug.

b Baseline was defined as the last non-missing measure prior to the first dose of the 3 times a day dosing regimen (i.e., Week 6).

**Fig. 2:**
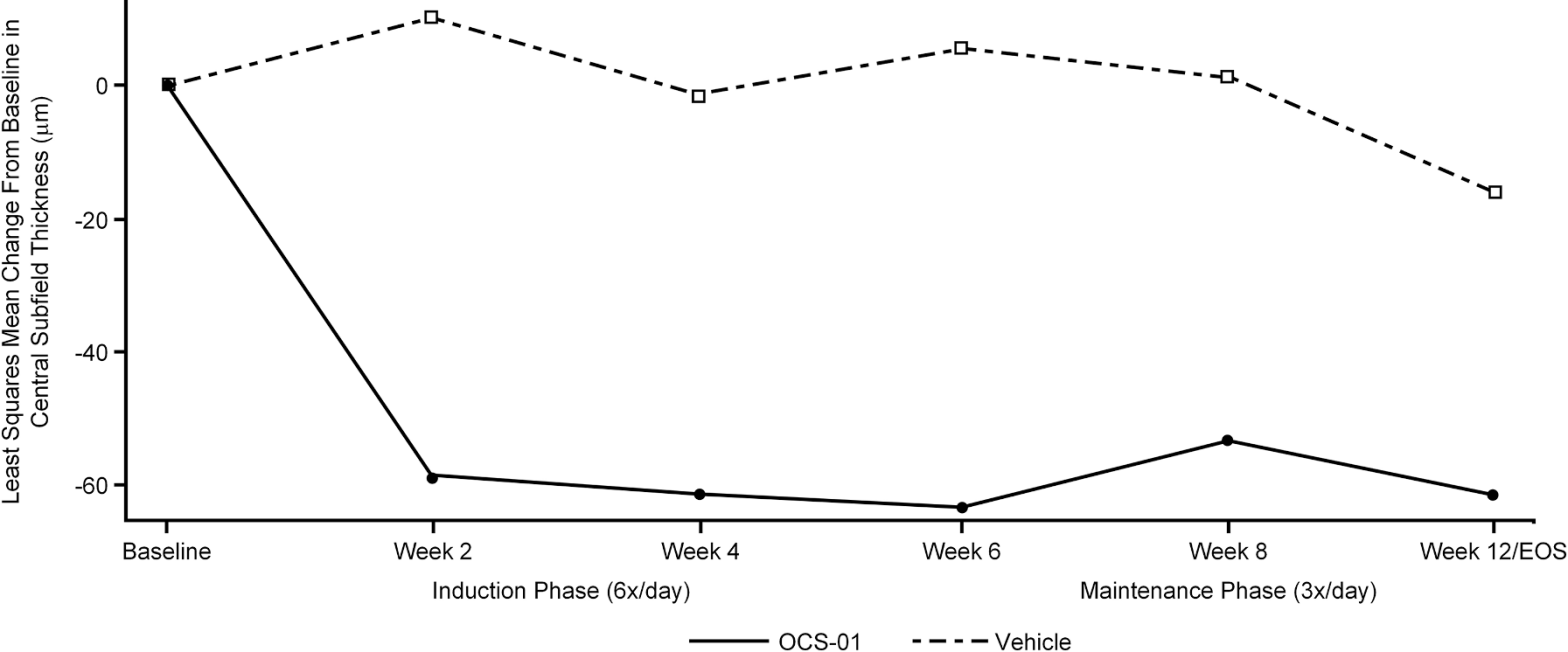
LS mean change in from baseline in CST (ITT Set). EOS = End of Study. Baseline is defined as the last non-missing measure prior to initiation of study treatment. Least squares means are from a mixed model for repeated measures using an Autoregressive 1 covariance structure, subject as a random effect, fixed effects for treatment and visit, the interaction between treatment and visit, and baseline central subfield thickness as a covariate. Solid line: OCS-01 group; dashed line: vehicle group.

Fig. 2 shows LS mean change in CST at each visit. A pronounced difference between treatment groups was already present at Week 2: mean changes from baseline were −61.1 ± 84.55 μm for the OCS-01 group and 12.5 ± 52.96 μm for the Vehicle group. A mixed model for repeated measures resulted in LS mean (SE) changes of −59.0 (8.39) μm in the OCS-01 group and 10.2 (12.17) μm in the Vehicle group, and an LS mean (SE) difference between treatment groups of −69.2 (14.80) μm at Week 2 compared with baseline (p < 0.0001). This treatment effect was sustained until Week 12. At Weeks 6 and 12, LS mean (SE) between-group differences in changes from baseline were −69.1 (14.6) μm and −45.6 (15.9) μm, respectively (Supplementary Table S6 in the Supplementary Appendix).

### Safety outcomes

In the OCS-01 group, 42/100 (42%) of patients experienced at least one ocular adverse event, as did 19/48 (40%) of the Vehicle group. Some differences between treatments in ocular adverse events in the study eye were apparent (Table 4): events relating to DMO were more common in the Vehicle group and events related to increased IOP were more common in the OCS-01 group. Other ocular adverse events showed similar frequencies in both groups.

**Table 4:** Ocular adverse events in the study, in 2 or more patients, by system organ class and preferred term (safety set).

System organ class Preferred term	n (%) patients		
	OCS-01 N = 100	Vehicle N = 48	Total N = 148
Any ocular TEAE in the study eye	42 (42.0)	19 (39.6)	61 (41.2)
Eye disorders	28 (28.0)	18 (37.5)	46 (31.1)
Diabetic retinal oedema	7 (7.0)	8 (16.7)	15 (10.1)
Ocular hypertension	8 (8.0)	0	8 (5.4)
Macular oedema	1 (1.0)	3 (6.3)	4 (2.7)
Vitreous haemorrhage	2 (2.0)	1 (2.1)	3 (2.0)
Cataract	1 (1.0)	1 (2.1)	2 (1.4)
Cataract cortical	1 (1.0)	1 (2.1)	2 (1.4)
Dry eye	2 (2.0)	0	2 (1.4)
Eyelid ptosis	2 (2.0)	0	2 (1.4)
Retinal exudates	1 (1.0)	1 (2.1)	2 (1.4)
Retinal thickening	1 (1.0)	1 (2.1)	2 (1.4)
Visual acuity reduced	1 (1.0)	1 (2.1)	2 (1.4)
Investigations	14 (14.0)	1 (2.1)	15 (10.1)
Intraocular pressure increased	14 (14.0)	1 (2.1)	15 (10.1)

Adverse events were coded using MedDRA version 26.0.

PT, preferred term; SOC, system organ class; TEAE, treatment-emergent adverse event.

Non-ocular adverse events were reported in 15/48 (31%) patients in the Vehicle group and 47/100 (47%) patients in the OCS-01 group (Supplementary Table S9 in the Supplementary Appendix). Three of 48 (6%) patients in the Vehicle group had serious adverse events (sepsis, cellulitis, and acute left ventricular failure, each in one patient) as did 5/100 (5%) patients in the OCS-01 group (dizziness, streptococcal pneumonia, abdominal pain upper and impaired gastric emptying, and hypertensive urgency). None of the serious adverse events were considered to be related to study medication. One patient in the OCS-01 group died: this was a 45-year-old female patient with type 1 diabetes and a recent history of hypertension who experienced a serious adverse event of severe hypertensive urgency (noted above) on study day 10 and was hospitalised. She was released from hospital 2 days later but died on study day 14. No autopsy data were available. The death was not considered to be related to study medication.

Intraocular pressure in the study eye is summarised by treatment group and visit in Table 5. Slightly higher mean IOP changes in the study eye from baseline were observed in the OCS-01 group (mean changes ranging from 0.64 to 2.79 mmHg) than the Vehicle group (mean changes ranging from −0.17 to 0.44 mmHg) at each visit. Mean changes from baseline within the OCS-01 group were similar from Week 4 onwards. None of the Vehicle group had an increase of at least 10 mmHg, but 16 patients (16%) in the OCS-01 group had such increases. There were nine patients in the OCS-01 group who had newly-occurring IOP values of 30 mmHg or higher; of these, the IOP decreased to at or below the baseline level by study end, except in those patients in which the 30 mmHg value occurred at the final study visit.

**Table 5:** Intraocular pressure in the study eye (safety set).

Visit	OCS-01 (N = 100)			Vehicle (N = 48)		
	Mean (SD)			Mean (SD)		
	n	Actual value	Change from baseline	n	Actual value	Change from baseline
Baseline	100	15.26 (3.050)		48	14.69 (2.962)	
Week 2	97	15.88 (3.434)	0.64 (2.846)	45	14.61 (2.686)	0.00 (2.759)
Week 4	96	17.61 (4.648)	2.39 (4.043)	45	14.71 (2.582)	−0.17 (3.073)
Week 6	95	16.87 (4.093)	1.61 (4.067)	41	15.15 (2.796)	0.44 (2.565)
Week 8	93	17.38 (4.754)	2.10 (4.305)	39	14.77 (3.191)	0.08 (2.834)
Week 12/EOS	84	18.13 (5.556)	2.79 (5.398)	40	14.60 (3.215)	−0.05 (2.710)
Increase from baseline ≥10 mmHg n (%)	16 (16%)			0 (−)		
Newly-occurring absolute value ≥ 30 mmHg at any visit n (%)	9 (9%)			0 (−)		
Any IOP lowering medication n (%)	22 (22%)			1 (2%)		

EOS, end of study. Baseline was defined as the last non-missing measure prior to initiation of study drug.

The between-group differences in IOP were reflected in rates of adverse events with preferred terms coding for IOP increases (intraocular pressure increased, and ocular hypertension, which together were reported for 22 patients in the OCS-01 group and 1 patient in the Vehicle group, Table 4). Among the patients with these adverse events in the OCS-01 group, the maximum severity was categorised as mild in 14 patients and moderate in eight. Two patients, both in the OCS-01 group, discontinued the study due to these adverse events; in one case the patient also had an adverse event of DMO that was involved in the decision to discontinue. In both cases the IOP elevations resolved after discontinuation. Eleven of the patients in the OCS-01 group who had adverse events related to IOP increases received topical treatment, as did two other patients in the OCS-01 group. No patient required surgery or laser treatment to control their IOP.

Regarding other ocular safety assessments, slit-lamp biomicroscopy, dilated indirect ophthalmoscopy or lens clarity grading assessments showed no notable between-group differences. No patients in either group required cataract surgery.

## Discussion

In Stage 1 of this randomised, double-masked, multicentre study, involving 148 participants, topical ocular OCS-01 dexamethasone ophthalmic suspension administered as a loading phase of six times/day for 6 weeks followed by a maintenance phase of 3 times/day improved visual acuity and decreased macular thickness relative to Vehicle in patients with DMO, and this regimen was adopted for Stage 2.

The improvement relative to Vehicle in visual acuity was demonstrated by the primary endpoint, change from baseline to Week 6 in BCVA ETDRS letter score, and secondary endpoints of proportion of patients with 15 letters or greater gain from baseline in BCVA ETDRS letters at Weeks 6 and 12, change in BCVA from baseline to Week 12, and AUC of BCVA ETDRS letters changes from baseline across post-baseline visits up to Weeks 6 and 12. Changes between Weeks 6 (loading phase) and 12 (maintenance phase) did not show a difference between treatment groups, but the improvement in visual acuity in the OCS-01 group was maintained after switching to less frequent dosing at Week 6. Improvements from baseline in CST were apparent in the OCS-01 group after 2 weeks of treatment, and were also sustained after the switch to less frequent dosing (Weeks 6–12).

The efficacy assessments in Stage 1 of this study were intended to guide the dose regimen in Stage 2. Stage 2 of the current study, in which patients will be treated for 1 year, should provide confirmatory data to compare with those from studies with intravitreal therapies. Mean BCVA gains with intravitreal VEGF inhibitors after treatment periods ranging from 12 to 60 months (most commonly 36 months) range from 6.7 to 14.2 letters,^22^ as compared with the treatment difference between OCS-01 and Vehicle of 4.1 letters after 6 weeks in the current study. Comparisons with studies using intravitreal therapies for DMO are difficult not only due to differences in study duration but also to variations in methodology.^5^^,^^12^^,^^23^, ^24^, ^25^ While the BCVA gains in this study at Weeks 6 and 12 with OCS-01 appear broadly comparable with those observed at the same time points in the RIDE and RISE studies with ranibizumab,^26^ and the proportion of patients with at least a 15-letter gain from baseline at 12 weeks with OCS-01 appeared to be higher than that at the same time point with a dexamethasone 700 μg implant (Ozurdex®),^27^ such comparisons should be interpreted with great caution.

OCS-01 was well-tolerated in this study; the main differences between active treatment and vehicle in adverse events were higher rates of events related to increased IOP in the OCS-01 group, and higher rates of events relating to the underlying condition in the vehicle group. Increased IOP is an expected complication of ocular corticosteroid treatment; the IOP increases observed with OCS-01 were in most cases relatively minor, however, and topical application should facilitate management of IOP increases, as unlike intravitreal implants, topical treatment can be discontinued easily.

Limitations of Stage 1 of this study are linked to its design and its objective, namely to test a dosing regimen for use in Stage 2, specifically estimating the magnitude of the treatment effect in terms of changes in BCVA. They include the 12-week duration, meaning that the longer-term magnitude and durability of the observed effects is unknown; Stage 2 of the study, with a 1-year duration will address this. Also, this was a Vehicle-controlled study rather than using an established treatment as an active control. The use of an intravitreal therapy as a control would have been complex in terms of masking (a double-dummy design would have been needed), and the use of sham intravitreal injections in patients assigned to topical treatment raises ethical concerns. As a result, however, comparison with the treatment effects observed with established therapies are difficult, as noted previously. Further limitations include the inclusion of both previously-treated and treatment-naïve patients without stratification during randomisation, and the lack of a subgroup analysis assessing the relative effects of treatment in these groups. In practice, the proportions of patients with previous anti-VEGF treatment were similar between groups, so it was unlikely that the lack of stratification would have influenced overall outcomes. Further limitations include the lack of subgroup analyses or multivariate statistical models based on other baseline characteristics, for example, BCVA, CST, phakic status, disease duration, and glycaemic control. Such analyses were not performed as Stage 1 had a relatively small patient population that might limit the usefulness of subgroup analysis. In addition, the study population was highly selected, particularly with respect to baseline visual acuity, and this limits the generalisation of the study results to the DMO population as a whole. The baseline visual acuity range for the study was chosen based on a subgroup analysis in a previous Phase 2 study with OCS-01, in which patients with BCVA <65 letters at baseline showed greater improvements in both BCVA and CMT than those with ≥65 letters.^20^ Finally, as patients with IOP >21 mmHg or suspected glaucoma were excluded from the study, the safety in terms of IOP changes may not be generalisable to the total population of patients with DMO.

The study population also showed some differences in baseline demographic characteristics to published data on patients with DMO. The mean age of patients was comparable with that in published data,^1^^,^^28^^,^^29^ but the proportion of male patients appeared to be slightly higher. The race distribution of patients in the study (who were predominantly Caucasian) also differed from that in the general population of patients with DMO, where, although the data vary between publications, there are a higher proportion of patients categorised as of Black or African American race than in our study population. This probably reflects the population attending the study centres, and might reduce the generalisability of the study results.

The circumstances of patients with DMO vary, in terms of symptoms and severity of the condition, access or otherwise to centres offering intravitreal therapies, and economic circumstances. Intravitreal therapies place a high burden on patients and health care systems, particularly with long-term use. If the findings of Stage 1 of this study are confirmed, and longer-term effectiveness established in Stage 2, effective topical therapy could complement existing intravitreal treatments, being non-invasive and not requiring specialist facilities and personnel. Well-tolerated topical therapy could be useful in patients with recent onset of DMO, with central involved DMO with good visual acuity (where observation has been demonstrated to be non-inferior to immediate initiation of intravitreal or laser treatment in prevention of vision loss at 2 years^30^), those with mild to moderate visual impairment who are not candidates for intravitreal treatment, or could be used prior to patients being able to access intravitreal therapy. Topical treatment would provide more options to tailor treatment to individual patients’ needs, and has the potential to widen access to treatment.

In conclusion, the results of Stage 1 of the current study suggest that topical OCS-01 is well-tolerated with no unexpected safety findings and may be effective in improving BCVA and CST in patients with DMO over the 12-week study duration; the confirmatory Stage 2 of this study will assess longer-term outcomes.

## Contributors

All authors contributed equally to the review and editing of the manuscript. A.M.K., R.T., S.P., H.S-H., and D.S.B. treated patients and contributed to data collection and to the review and interpretation of the data. S.M. contributed to the study design and data review and interpretation. All authors had full access to the data in the study and had final responsibility for the decision to publish. A.M.K. and S.M. directly accessed and verified the underlying data reported here. Phil Hunt, of Southdown Medical Writing Ltd, funded by Oculis, assisted with the preparation of the manuscript.

## Data sharing statement

It is not planned to share individual participant data. Other study documents (protocol, informed consent document, and statistical analysis plan) will be shared on ClinicalTrials.gov (NCT05066997) on completion of the study.

## Declaration of interests

A.M.K. reports grants or contracts from Aviceda, Adverum, Alexion, Annexon, Apellis Pharmaceuticals, Astellas, Aviceda Therapeutics, Complement Therapeutics, 4DMT, Eyepoint Pharmaceuticals, Exegenesis, Genentech, Gyroscope Therapeutics, Iveric Bio, Janssen, Kodiak, Kyowa Kirin, Neurotech, Ocular Therapeutix, Oxular, Regenxbio, Roche, Sanofi, and Vanot; consulting fees from Abbvie, ADARx Pharmaceuticals, Adverum, Alcon, Alkeus, Allgenesis, Amgen, Annexin, Annexon, Apellis Pharmaceuticals, Ashvattha, Therapeutics, Astellas, Aviceda Therapeutics, Beacon Therapeutics, Boehringer Ingelheim, Clearside Biomedical, Complement Therapeutics, 4DMT, Exegenesis, EyePoint Pharmaceuticals, Fronterra Therapeutics, Genentech, Gyroscope Therapeutics, Harrow, i-Lumen Scientific, InFocus, Iveric Bio, Janssen Pharmaceuticals, Kodiak Sciences, Kriya Therapeutics, Kyowa Kirin, Merit, Neurotech, Nanoscope, Novartis, Ocular Therapeutix, Oculis, Ocuphire, Ocuterra, Ollin, Olive BioPharma, Opthea, Opus Genetics, Oxular, Oxurion, Perfuse, Ray Therapeutics, Recens Medical, Regeneron Pharmaceuticals, Regenxbio, Revive, RevOpsis, Roche, Samsung, Sanofi, Stealth BioTherapeutics, Surrozen, Thea Pharma, Therini, Unity Biotechnology, Vanotech, Vial, and ZipBio; and holds stock or stock options in Ashvattha, Aviceda Therapeutics, Oculis, Ollin, Opthea, PolyPhotonix, Recens Medical, Perfuse, RevOpsis, Vial, and ZipBio.

On behalf of R.T. (deceased), the corresponding author reports fees from Abbvie/Allergan, Alcon, Apellis, Bayer, Boehringer Ingelheim, Genentech, Iveric Bio, Novartis, Oculis, Roche, Thea, and Zeiss.

D.S.B. reports consulting fees from 4DMT, AbbVie, ADARx, Adverum Bio, AiViva, Alcon, Aldeyra, Alkahest, Allegro, Allgenesis, Alumis, Amgen, Amaros, Annexon Bio, Apellis, AGTC, Ashvattha, Astellas, Aviceda, Bausch & Lomb, Bayer, Belite Bio, BioCryst, Bionic Vision, Biovisics, Boehringer-Ingelheim, Clearside, EyePoint, Frontera, Genentech, Glaukos, Janssen, jCyte, Kriya, Kyowa Kirin, Lineage Cell, LumiThera, Nanoscope, Neurotech, Novartis, Ocugen, Oculis, Ocuphire, OcuTerra, Ocutrx Vision, Opthea, Optos, Ora, Palatin, Pfizer, Perceive Bio, Ray Therap, Regeneron, RegenxBio, Rezolute, Ripple, Samsung Bioepis, Sanofi, Science Branding, Smilebiotek, Stealth Bio, Sun Pharma, Thea, Unity bio, Valo, Vanotech, Visgenx, Viranu, Vitro Bio, Viva Vision.

S.P. reports grants or contracts from Aerie, Aerpio, Allergan, Allgenesis, Apellis, Boehringer lngelheim, Chengdu Kanghong, Clearside, EyePoint, Genentech-Roche, lonis Pharmaceuticals, IVERIC Bio, KalVista, Kodiak Sciences, Mylan, Novartis, Oculis, Opthea, Ora, Oxurion, Regeneron, Samsung, SmileBiotek, Stealth Biotherapeutics, ThromboGenics, and Xbrane Biopharma; consulting fees from AiViva, Allergan, Allgenesis, Genentech- Roche, Kala, Kodiak Sciences, Ocugenix, RegenxBio; participation on a Data Safety Monitoring Board or Advisory Board for Ocugenix, Allergan, Allgenesis Biotherapeutics, Genentech-Roche, Kodiak Sciences, and lveric Bio; and holds stock or stock options in Allgenesis.

H.S-H. reports no conflicts of interest.

S.M. is a consultant to Oculis, the study sponsor.

## References

[1] Yau J.W., Rogers S.L., Kawasaki R. (2012). Global prevalence and major risk factors of diabetic retinopathy. Diabetes Care.

[2] Early Treatment Diabetic Retinopathy Study Research Group (1985). Photocoagulation for diabetic macular edema. Arch Ophthalmol.

[3] Browning D.J., Stewart M.W., Lee C. (2018). Diabetic macular edema: evidence-based management. Indian J Ophthalmol.

[4] Nguyen Q.D., Brown D.M., Marcus D.M. (2012). Ranibizumab for diabetic macular edema: results from 2 phase III randomized trials: RISE and RIDE. Ophthalmology.

[5] Elman M.J., Bressler N.M., Qin H. (2011). Expanded 2-year follow-up of ranibizumab plus prompt or deferred laser or triamcinolone plus prompt laser for diabetic macular edema. Ophthalmology.

[6] Do D.V., Nguyen Q.D., Boyer D. (2012). One-year outcomes of the da Vinci Study of VEGF Trap-Eye in eyes with diabetic macular edema. Ophthalmology.

[7] Sharma Y.R., Tripathy K., Venkatesh P., Gogia V. (2014). Aflibercept: how does it compare with other anti-VEGF drugs. Austin J Clin Ophthalmol.

[8] Sahni J., Patel S.S., Dugel P.U. (2019). Simultaneous inhibition of angiopoietin-2 and vascular endothelial growth factor-A with faricimab in diabetic macular edema: BOULEVARD phase 2 randomized trial. Ophthalmology.

[9] Wykoff C.C., Abreu F., Adamis A.P. (2022). Efficacy, durability, and safety of intravitreal faricimab with extended dosing up to every 16 weeks in patients with diabetic macular oedema (YOSEMITE and RHINE): two randomised, double-masked, phase 3 trials. Lancet.

[10] Brown D.M., Emanuelli A., Bandello F. (2022). KESTREL and KITE: 52-week results from two phase III pivotal trials of brolucizumab for diabetic macular edema. Am J Ophthalmol.

[11] Glassman A.R., Wells I.I.I.J.A., Josic K. (2020). Five-year outcomes after initial aflibercept, bevacizumab, or ranibizumab treatment for diabetic macular edema (Protocol T Extension Study). Ophthalmology.

[12] Boyer D.S., Yoon Y.H., Belfort R. (2014). Three-year, randomized, sham-controlled trial of dexamethasone intravitreal implant in patients with diabetic macular edema. Ophthalmology.

[13] Campochiaro P.A., Brown D.M., Pearson A. (2012). Sustained delivery fluocinolone acetonide vitreous inserts provide benefit for at least 3 years in patients with diabetic macular edema. Ophthalmology.

[14] Campochiaro P.A., Brown D.M., Pearson A. (2011). Long-term benefit of sustained-delivery fluocinolone acetonide vitreous inserts for diabetic macular edema. Ophthalmology.

[15] International Diabetes Federation (2019).

[16] Bakri S.J., Wolfe J.D., Regillo C.D., Flynn H.W., Wykoff C.C. (2019). Evidence-based guidelines for management of diabetic macular edema. J Vitreoretinal Dis.

[17] Schmidt-Erfurth U., Garcia-Arumi J., Bandello F. (2017). Guidelines for the management of diabetic macular edema by the European Society of retina specialists (EURETINA). Ophthalmologica.

[18] del Amo E.M., Rimpelä A.K., Heikkinen E. (2017). Pharmacokinetic aspects of retinal drug delivery. Progress Ret Eye Res.

[19] Loftsson T., Stefánsson E. (2022). Nanoparticle eye drops deliver therapeutic drug concentrations to both anterior and posterior segment. Acta Ophthalmol.

[20] Stefánsson E., Loftsson T., Larsen M. (2023). Topical treatment of diabetic macular edema using dexamethasone ophthalmic suspension: a randomized, double-masked, vehicle-controlled study. Acta Ophthalmol.

[21] Chew E.Y., Kim J., Sperduto R.D. (2010). Evaluation of the age-related eye disease study clinical lens grading system AREDS report No. 31. Ophthalmology.

[22] Cheema A.A., Cheema H.R. (2024). Diabetic macular edema management: a review of anti-vascular endothelial growth factor (VEGF) therapies. Cureus.

[23] The Diabetic Retinopathy Clinical Research Network (2015). Aflibercept, bevacizumab, or ranibizumab for diabetic macular edema. N Engl J Med.

[24] Bressler N.M., Beaulieu W.T., Maguire M.G. (2018). Early response to anti-vascular endothelial growth factor and two-year outcomes among eyes with diabetic macular edema in Protocol T. Am J Ophthalmol.

[25] Elman M.J., Aiello L.P., Beck R.W. (2010). Randomized trial evaluating ranibizumab plus prompt or deferred laser or triamcinolone plus prompt laser for diabetic macular edema. Ophthalmology.

[26] Food and Drugs Administration Lucentis. Full prescribing information. https://www.accessdata.fda.gov/drugsatfda_docs/label/2014/125156s105lbl.pdf.

[27] Food and Drugs Administration Ozurdex. Full prescribing information. https://www.accessdata.fda.gov/drugsatfda_docs/label/2014/022315s009lbl.pdf.

[28] Haliyur R., Marwah S., Mittal S. (2024). Demographic and metabolic risk factors associated with development of diabetic macular edema among persons with diabetes mellitus. Ophthalmol Sci.

[29] Varma R., Bressler N.M., Doan Q.V. (2014). Prevalence of and risk factors for diabetic macular edema in the United States. JAMA Ophthalmol.

[30] Baker C.W., Glassman A.R., Beaulieu W.T. (2019). Laser photocoagulation vs observation on vision loss among patients with diabetic macular edema involving the center of the macula and good visual acuity. a randomized clinical trial. JAMA.

