# Supplementary material for: Safety and efficacy of topical OCS-01 ophthalmic suspension for the treatment of diabetic macular oedema: stage 1 of a multicentre, double-blind, randomised, vehicle-controlled phase 2/3 trial
Source: eClinicalMedicine. 2026 Jul 27;98:104091. doi: 10.1016/j.eclinm.2026.104091 (PMC13445531; doi:10.1016/j.eclinm.2026.104091)
Supplement: Supplementary file 1 [file mmc1.pdf]

Arshad M. Khanani, Ramin Tadayoni (deceased), Sabri Markabi, Sunil Patel, Hani Salehi-Had, and David S. Boyer.

Safety and efficacy of topical OCS-01 ophthalmic suspension for the treatment of diabetic macular oedema; stage 1 of a multicentre, double-blind, randomised, vehicle-controlled phase 2/3 trial: Supplementary Appendix

Table of contents

Full list of study inclusion and exclusion criteria 3

Ethics committee approvals 6

Table S1 Sensitivity analyses change in BCVA ETDRS letter score from baseline to Week 6: (ITT Set) 7

Table S2 Proportions of patients with a 10 letter/2 line or greater gain from baseline in ETDRS BCVA at each visit (ITT Set) 8

Table S3 Proportions of patients with a 5 letter/1 line or greater gain from baseline in ETDRS BCVA at each visit (ITT Set) 9

Table S4 Secondary efficacy analyses based on changes from Baseline to Week 12 in BCVA (ITT Set). 10

Table S5 Analysis of 3-Line or Greater Gain in BCVA assessment from Baseline at each follow-up visit (ITT Set) 11

Table S6 Changes from baseline in CST (ITT Set) 12

Table S7 Non-ocular adverse events in 2 or more patients at by system organ class and preferred term (Safety Set) 13

Figure S1 Change in BCVA ETDRS letter score from baseline to Week 12 (ITT Set) 14

Full inclusion and exclusion criteria:

Inclusion Criteria

To participate in Stage 1 of the study, patients must have met the following criteria:

1. Have a signed ICF before any study-specific procedures were performed.
2. Be a male or female adult patient aged 18 to 85 years. Race and ethnicity information was collected based on National Institutes of Health criteria.
3. Have DME with presence of intraretinal and/or subretinal fluid in the study eye, with CST of ≥310 µm (may have been adjusted based on gender specific requirements) by SD-OCT at screening (Visit 1) (as assessed by an IRC); CST was not part of the eligibility reconfirmation on Day 1.
4. Participants required BCVA ETDRS letter score of >34 letters (>20/200 Snellen equivalent) in the non-study eye at screening (ie, as per definition of monocular blindness).
5. Have a BCVA ETDRS letter score of ≤65 (Snellen 20/50) and ≥24 (Snellen 20/320) in the study eye at screening and baseline (Visit 1 and Visit 2).
6. Have a documented diagnosis of type 1 or type 2 diabetes mellitus and a glycosylated HbA1c of ≤12.0% (≤108 mmol/mol) at Visit 1 (screening).

Clarification: For inclusion criteria 7 and 8, each patient must have met 1 criterion or the other.

7. Participants who had been treated with any anti-VEGF agents IVT and/or corticosteroids periocular or IVT in the study eye in the past and for whom the following washout periods before Day 1 would apply:
   a. Anti-VEGF agents IVT: 3 months.
   b. Periocular or IVT corticosteroids:
      i. Triamcinolone: 4 months
      ii. Biodegradable slow-release steroid IVT implant (eg, Ozurdex): 6 months
      iii. Nonbiodegradable slow-release steroid implant (eg, Iluvien, Retisert, Yutiq): 3 years
8. Participants who were anti-VEGF and corticosteroid IVT treatment-naïve (ie, have not received previous treatment with any anti-VEGF and corticosteroid IVT) in the study eye.
9. Have a negative urine pregnancy test at Visit 1, if WOCBP (those who have experienced menarche and who are not surgically sterilized [bilateral tubal ligation, hysterectomy, or bilateral oophorectomy] or postmenopausal [12 months after last menses]) and must have used adequate birth control throughout the study period.

Exclusion Criteria

To participate in Stage 1 of the study, patients must not have met any of the following criteria: OCULAR

1. Have macular edema considered to be because of a cause other than DME.

   Note: an eye was not considered eligible if: (1) the macular edema was considered to be related to ocular surgery such as cataract extraction;(2) clinical examination and/or OCT suggested that vitreoretinal interface abnormalities disease (eg, a taut posterior hyaloid or epiretinal membrane) was the primary cause of the macular edema, or (3) the macular edema was considered to be related to another condition such as age-related macular degeneration, uveitis, retinal vein occlusion, or drug toxicity. If the DME consisted of circumscribed, focal leakage that the evaluating Investigator believed should be treated with laser and no other treatments, the eye was not eligible to be a study eye.

2. Have a decrease in BCVA because of causes other than DME (eg, foveal atrophy, pigment abnormalities, dense subfoveal hard exudates, previous vitreoretinal surgery, central serous retinopathy, nonretinal condition, substantial cataract, macular ischemia) that, in the Investigator's opinion, was likely to be decreasing BCVA by 3 lines or more (ie, cataract would be reducing acuity to 20/40 or worse if eye was otherwise normal) in the study eye.
3. Have a known history of significant macular ischemia which would have prevented gain in VA in the study eye.
4. Have any other ocular disease that may have caused substantial reduction in BCVA, including retinal detachment, epiretinal membrane, vitreous hemorrhage or fibrosis involving the macula in the study eye, ocular inflammation (uveitis), other retinal inflammatory or infectious diseases.
5. Have active or suspected periocular or ocular infection in the study eye (eg, keratitis, scleritis, or conjunctivitis). Mild noninfectious blepharitis was accepted.
6. Have a history of noninfectious uveitis in the study eye.
7. History of herpetic ocular disease in the study eye.
8. Hazy ocular media in the study eye, as assessed by the Investigator, that may have affected fundus examination and OCT measurements.
9. IOP >21 mm Hg at Screening in the study eye (glaucoma suspects).
10. History of glaucoma and/or steroid induced elevated IOP in the either eye.
11. Cup to disc ratio more than 0.5 in either eye.
12. Patients who planned to continue using contact lenses as a main source of vision correction during the study in the study eye.
13. Patients who planned to use cosmetic contact lenses in the study eye during the study.
14. History of major intraocular ocular surgery within 3 months including cataract surgery in the study eye.
15. Panretinal photocoagulation in the study eye; OR grid laser within 1000 microns of the foveal center in the study eye.

SYSTEMIC

16. Participants who were currently enrolled in or had participated in any other clinical study involving an investigational product or device, or in any other type of medical research, within 30 days before screening and up to completion of the current study.
17. Systemic corticosteroids (prednisone or methylprednisolone) either oral or injectable were not allowed in the study. The use of acute inhaled corticosteroids was permissible during the study for up to 14 days.
18. Any prior or concomitant systemic anti-VEGF treatment within 6 months before Day 1.
19. Significant medical conditions that in the opinion of the Investigator may have affected the patient compliance with study visits.
20. Female participants must not have been pregnant or breastfeeding.
21. WOCBP who were not able to comply with adequate birth control throughout the study period.

22. History of chronic renal failure that required dialysis or kidney transplant.

Study eye:

Eyes were eligible for analysis if they met all of the inclusion criteria and none of the exclusion criteria (Clarification: for inclusion criteria 7 and 8, each patient had to meet 1 criterion or the other). If both eyes were eligible for analysis, the study eye was the eye with the worst BCVA ETDRS letters score at Day 1. If both eyes had the same BCVA ETDRS letters score at Day 1, the right eye was considered the study eye.

Ethics committee approvals

Study centre	Ethics committee	Date of approval	IRB/IEC site reference number
Verum Research, LLC, Eugene, Oregon, USA	Advarra, Columbia, Maryland, USA	13/09/2021	SSU00164526
Retina-Vitreous Associates Medical Group, Beverly Hills, California, USA	Advarra, Columbia, Maryland, USA	15/09/2021	SSU00162348
Cumberland Valley Retina Consultants, Hagerstown, Maryland, USA	Advarra, Columbia, Maryland, USA	21/10/2021	SSU00167989
Southeast Retina Center, P.C., Carmel, Indiana, USA	Advarra, Columbia, Maryland, USA	07/09/2021	SSU00163527
Southeast Retina Center, P.C., August, Georgia, USA	Advarra, Columbia, Maryland, USA	23/09/2021	SSU00163950
Medical Center Ophthalmology Associates, San Antonio, Texas, USA	Advarra, Columbia, Maryland, USA	29/09/2021	SSU00166045
Fort Lauderdale Eye Institute, Plantation, Florida, USA	Advarra, Columbia, Maryland, USA	13/10/2021	SSU00167254
Center for Retina care at the Eye Institute, Melbourne, Florida, USA	Advarra, Columbia, Maryland, USA	17/09/2021	SSU00164759
Retina Research Institute of Texas Integrated Clinical Research, Abilene, Texas, USA	Advarra, Columbia, Maryland, USA	16/11/2021	SSU00169786
Marietta Eye Clinic, Marietta, Georgia, USA	Advarra, Columbia, Maryland, USA	08/09/2021	SSU00164014
Salehi Retina Institute dba Retina Associates of Southern California, Huntington Beach, California, USA	Advarra, Columbia, Maryland, USA	17/11/2021	SSU00169352
MedEye Associates, Miami, Florida, USA	Advarra, Columbia, Maryland, USA	10/11/2021	SSU00169575
Black Hills Regional Eye Institute, Rapid City, South Dakota, USA	Advarra, Columbia, Maryland, USA	16/09/2021	SSU00164971
Sarasota Retina Institute, Sarasota, Florida, USA	Advarra, Columbia, Maryland, USA	15/12/2021	SSU00172561
Retina-Vitreous Surgeons of Central New York (RVSCNY), PC - Syracuse Location, Liverpool, New York, USA	Advarra, Columbia, Maryland, USA	13/10/2021	SSU00167282
Austin Retina Associates, Round Rock, Texas, USA	Advarra, Columbia, Maryland, USA	28/09/2021	SSU00165490
Arizona Retina and Vitreous Consultants, Phoenix, Arizona, USA	Advarra, Columbia, Maryland, USA	20/10/2021	SSU00168018
Sterling Research Group, Cincinnati, Ohio, USA	Advarra, Columbia, Maryland, USA	16/11/2021	SSU00170172
Sierra Eye Associates, Reno, Nevada, USA	Advarra, Columbia, Maryland, USA	21/01/2022	SSU00175238
Valley Retina Institute P.A. VRI – McAllen, McAllen, Texas, USA	Advarra, Columbia, Maryland, USA	01/12/2021	SSU00171480
Retina Associates, Elmhurst, Illinois, USA	Advarra, Columbia, Maryland, USA	01/12/2021	SSU00171385
Florida Retina Institute, Orlando, Florida, USA	Advarra, Columbia, Maryland, USA	21/12/2021	SSU00172644
Advanced Research, Coral Springs, Florida, USA	Advarra, Columbia, Maryland, USA	15/03/2022	SSU00179724
Retinal Research Institute LLC, Phoenix, Arizona, USA	Advarra, Columbia, Maryland, USA	26/05/2022	SSU00186281
Mid Atlantic Retina Specialists, Hagerstown, Maryland	Advarra, Columbia, Maryland, USA	06/05/2022	SSU00184391
The Retina Institute, Saint Louis, Missouri, USA	Advarra, Columbia, Maryland, USA	23/06/2022	SSU00188212
California Eye Specialists Medical Group, Pasadena, California, USA	Advarra, Columbia, Maryland, USA	12/07/2022	SSU00189122
Island Retina, Shirley, New York, USA	Advarra, Columbia, Maryland, USA	02/06/2022	SSU00185981
Retina Center of Texas, Southlake, Texas, USA	Advarra, Columbia, Maryland, USA	01/06/2022	SSU00186572
The Retina Partners, Encino, California, USA	Advarra, Columbia, Maryland, USA	23/08/2022	SSU00193988
Blue Ocean Clinical Research, Clearwater, Florida, USA	Advarra, Columbia, Maryland, USA	17/06/2022	SSU00186812
Piedmont Eye Center, Lynchburg, Virginia, USA	Advarra, Columbia, Maryland, USA	03/08/2022	SSU00191224
North Carolina Retina Associates, Wake Forest, North Carolina, USA	Advarra, Columbia, Maryland, USA	01/08/2022	SSU00191005
San Antonio Eye Center- Downtown, San Antonio, Texas, USA	Advarra, Columbia, Maryland, USA	03/08/2022	SSU00189484
Retina Partners of Northwest Arkansas, Springdale, Arkansas, USA	Advarra, Columbia, Maryland, USA	01/08/2022	SSU00192179
MH EK, Budapest, Hungary	Klinikai Farmakológiai Etikai Bizottsága (KFEB), Budapest, Hungary	10/08/2022	005199
Ganglion Medical Center, Pecs, Hungary	Klinikai Farmakológiai Etikai Bizottsága (KFEB), Budapest, Hungary	10/08/2022	005199
DE KK, Debrecen, Hungary	Klinikai Farmakológiai Etikai Bizottsága (KFEB), Budapest, Hungary	10/08/2022	005199
Budapest Retina Associates, Budapest, Hungary	Klinikai Farmakológiai Etikai Bizottsága (KFEB), Budapest, Hungary	10/08/2022	005199

IRB: institutional review board; IEC: independent ethics committee. Information provided for all sites that screened patients

Table S1 Sensitivity analyses change in BCVA ETDRS letter score from baseline to Week 6 (ITT Set)

Visit	Statistics	Vehicle (N=48) Actual Value	Vehicle (N=48) Change From Baseline	OCS-01 (N=100) Actual Value	OCS-01 (N=100) Change From Baseline
Baseline	Mean (SD)	58·3 (7·51)		57·5 (9·26)	
Sensitivity analysis 1- mixed model for repeated measures[a]					
Week 6	LS mean (SE)		3·1 (1·24)		7·2 (0·84)
	95% CI		(0·6, 5·5)		(5·6, 8·9)
	LS mean difference (OCS-01 - Vehicle) (SE)				4·2 (1·50)
	95% CI				(1·2, 7·1)
	p-value				0·006
Sensitivity analysis 2 - mixed model for repeated measures[b]					
Week 6	LS mean (SE)		3·2 (1·26)		7·1 (0·84)
	95% CI		(0·8, 5·7)		(5·4, 8·7)
	LS mean difference (OCS-01 - Vehicle) (SE)				3·8 (1·53)
	95% CI				(0·8, 6·8)
	p-value				0·013
Sensitivity analysis 3 - mixed model for repeated measures[c]					
Week 6	LS mean (SE)		3·1 (1·25)		7·3 (0·85)
	95% CI		(0·7, 5·6)		(5·6, 8·9)
	LS mean difference (OCS-01 - Vehicle) (SE)				4·1 (1·51)
	95% CI				(1·2, 7·1)
	p-value				0·007

Abbreviations: BCVA, best corrected visual acuity; ETDRS, Early Treatment Diabetic Retinopathy Study; ITT, intent-to-treat; LS, least squares.

Notes: Baseline was defined as the last non-missing measure prior to initiation of study drug. In all 3 sensitivity analyses, change from baseline compared between treatment groups was analysed with a mixed model for repeated measures using an Autoregressive 1 covariance structure, subject as a random effect, fixed effects for treatment and visit, the interaction between treatment and visit, and baseline BCVA ETDRS letter score as a covariate.

[a] All missing data were assumed to be missing at random (treatment-based regression methodology). Table header N's indicate the number of subjects contributing data to the statistical modelling.

[b] All missing data were assumed to be missing not at random (vehicle-based regression methodology). Table header N's indicate the number of subjects contributing data to the statistical modelling.

[c] Only available data were used in the model (no imputation). A total of 41 subjects in the Vehicle group and 95 subjects in the OCS-01 group contributed data to the statistical modelling.

Table S2 Proportions of patients with a 10 letter/2 line or greater gain from baseline in ETDRS BCVA at each visit (ITT Set)

Visit	Statistics	OCS-01 (N=100)	Vehicle (N=48)
Week 2	n	97	45
	≥10 letters gain (response), n (%)	25 (25·8)	6 (13.3)
	<10 letters gain, n (%)	72 (74·2)	39 (86.7)
	Estimated marginal effects of treatment	25·67	13.62
	95% CI	17·03, 34·31	3.58, 23.67
	Difference in marginal effects (OCS-01 – Vehicle)	12·05	
	95% CI	-1·22, 25·31	
	p-value	0·075	
	Adjusted odds ratio (OCS-1/Vehicle)	2·20	
	95% Wald CI for odds ratio	0·83, 5·80	
	p-value	0·112	
Week 4	n	96	45
	≥10 letters gain (response), n (%)	34 (35·4)	10 (22.2)
	<10 letters gain, n (%)	62 (64·6)	35 (77.8)
	Estimated marginal effects of treatment	35·24	21.36
	95% CI	25·71, 44·77	9.60, 33.12
	Difference in marginal effects (OCS-01 – Vehicle)	13·88	
	95% CI	-1·25, 29·01	
	p-value	0·072	
	Adjusted odds ratio (OCS-1/Vehicle)	2·01	
	95% Wald CI for odds ratio	0·89, 4·54	
	p-value	0·095	
Week 6	n	95	41
	≥10 letters gain (response), n (%)	39 (41·1)	9 (22.0)
	<10 letters gain, n (%)	56 (58·9)	32 (78.0)
	Estimated marginal effects of treatment	40·36	22.07
	95% CI	30·54, 50·18	9.74, 34.40
	Difference in marginal effects (OCS-01 – Vehicle)	18·30	
	95% CI	2·46, 34·13	
	p-value	0·024	
	Adjusted odds ratio (OCS-1/Vehicle)	2·40	
	95% Wald CI for odds ratio	1·04, 5·51	
	p-value	0·039	
Week 8	n	93	39
	≥10 letters gain (response), n (%)	39 (41·9)	10 (25.6)
	<10 letters gain, n (%)	54 (58·1)	29 (74.4)
	Estimated marginal effects of treatment	40·56	24.81
	95% CI	30·77, 50·36	11.83, 37.79
	Difference in marginal effects (OCS-01 – Vehicle)	15·75	
	95% CI	-0·55, 32·05	
	p-value	0·058	
	Adjusted odds ratio (OCS-1/Vehicle)	2·08	
	95% Wald CI for odds ratio	0·92, 4·67	
	p-value	0·077	
Week 12/EOS	n	84	40
	≥10 letters gain (response), n (%)	41 (48·8)	11 (27.5)
	<10 letters gain, n (%)	43 (51·2)	29 (72.5)
	Estimated marginal effects of treatment	43·99	26.56
	95% CI	33·83, 54·14	13.27, 39.85
	Difference in marginal effects (OCS-01 – Vehicle)	17·43	
	95% CI	0·67, 34·18	
	p-value	0·042	
	Adjusted odds ratio (OCS-1/Vehicle)	2·18	
	95% Wald CI for odds ratio	0·98, 4·85	
	p-value	0·057	

EOS = End of Study. Baseline is defined as the last non-missing measure prior to initiation of study treatment. Model used is a generalized estimating equation for binary response model with fixed effects for treatment and visit, the interaction between treatment and visit, and baseline BCVA ETDRS letter score as a covariate. Estimated marginal effects, difference in estimated marginal effects, and confidence interval limits from analysis are multiplied by 100. Imputation rules are applied based on a pattern-mixture model approach. Table header N's indicate the number of patients contributing data to the statistical modelling.

Table S3 Proportions of patients with a 5 letter/1 line or greater gain from baseline in ETDRS BCVA at each visit (ITT Set)

Visit	Statistics	OCS-01 (N=100)	Vehicle (N=48)
Week 2	n	97	45
	≥5 letters gain (response), n (%)	51 (52·6)	19 (42.2)
	<5 letters gain, n (%)	46 (47·4)	26 (57.8)
	Estimated marginal effects of treatment	52·68	41.98
	95% CI	42·86, 62·49	28.11, 55.85
	Difference in marginal effects (OCS-01 – Vehicle)	10·70	
	95% CI	-6·30, 27·69	
	p-value	0·217	
	Adjusted odds ratio (OCS-1/Vehicle)	1·54	
	95% Wald CI for odds ratio	0·77, 3·11	
	p-value	0·223	
Week 4	n	96	45
	≥ 5 letters gain (response), n (%)	51 (53·1)	21 (46.7)
	<5 letters gain, n (%)	45 (46·9)	24 (53.3)
	Estimated marginal effects of treatment	53·22	44.66
	95% CI	43·33, 63·10	30.59, 58.72
	Difference in marginal effects (OCS-01 – Vehicle)	8·56	
	95% CI	-8·56, 25·68	
	p-value	0·327	
	Adjusted odds ratio (OCS-1/Vehicle)	1·41	
	95% Wald CI for odds ratio	0·70, 2·84	
	p-value	0·330	
Week 6	n	95	41
	≥5 letters gain (response), n (%)	59 (62·1)	17 (41.5)
	<5 letters gain, n (%)	36 (37·9)	24 (58.5)
	Estimated marginal effects of treatment	61·49	41.39
	95% CI	51·77, 71·20	26.80, 55.99
	Difference in marginal effects (OCS-01 – Vehicle)	20·09	
	95% CI	2·49, 37·69	
	p-value	0·025	
	Adjusted odds ratio (OCS-1/Vehicle)	2·28	
	95% Wald CI for odds ratio	1·09, 4·78	
	p-value	0·029	
Week 8	n	93	39
	≥ 5 letters gain (response), n (%)	57 (61·3)	18 (46.2)
	<5 letters gain, n (%)	36 (38·7)	21 (53.8)
	Estimated marginal effects of treatment	60·23	45.12
	95% CI	50·22, 70·25	29.58, 60.65
	Difference in marginal effects (OCS-01 – Vehicle)	15·12	
	95% CI	-3·22, 33·45	
	p-value	0·106	
	Adjusted odds ratio (OCS-1/Vehicle)	1·86	
	95% Wald CI for odds ratio	0·87, 3·96	
	p-value	0·110	
Week 12/EOS	n	84	40
	≥ 5 letters gain (response), n (%)	59 (70·2)	20 (50.0)
	<5 letters gain, n (%)	25 (29·8)	20 (50.0)
	Estimated marginal effects of treatment	65·72	48.38
	95% CI	55·81, 75·64	33.18, 63.58
	Difference in marginal effects (OCS-01 – Vehicle)	17·34	
	95% CI	-1·00, 35·69	
	p-value	0·064	
	Adjusted odds ratio (OCS-1/Vehicle)	2·06	
	95% Wald CI for odds ratio	0·95, 4·46	
	p-value	0·065	

EOS = End of Study. Baseline is defined as the last non-missing measure prior to initiation of study treatment. Model used is a generalized estimating equation for binary response model with fixed effects for treatment and visit, the interaction between treatment and visit, and baseline BCVA ETDRS letter score as a covariate. Estimated marginal effects, difference in estimated marginal effects, and confidence interval limits from analysis are multiplied by 100. Imputation rules are applied based on a pattern-mixture model approach. Table header N's indicate the number of patients contributing data to the statistical modelling.

Table S4 Secondary efficacy analyses based on changes from Baseline to Week 12 in BCVA (ITT Set)

		OCS-01 (N=100)		Vehicle (N=48)	
Visit	Statistics	Actual value	Change	Actual value	Change
Change in BCVA ETDRS letter score at Week 12 compared with Baseline[a]					
Baseline	n	100		48	
	Mean (SD)	57·5 (9·26)		58·3 (7·51)	
Week 12/EOS	n	84	84	40	40
	Mean (SD)	66·6 (11·41)	8·7 (8·78)	62·7 (11·10)	3·9 (8·62)
	Mixed model for repeated measures[b]				
	LS mean (SE)		7·6 (0·89)		3·7 (1·35)
	95% CI		(5·8, 9·3)		(1·1, 6·4)
	LS mean difference (OCS-01 – Vehicle) (SE)		3·9 (1·61)		
	95% CI		(0·7, 7·0)		
	p-value		0·016		
Change in BCVA ETDRS Letter Score at Week 12 Compared With Week 6[c]					
Week 6	n	93		40	
	Mean (SD)	65·0 (12·65)		62·3 (11·06)	
Week 12/EOS	n	84	84	39	39
	Mean (SD)	66·6 (11·41)	0·7 (5·17)	63·0 (11·08)	0·7 (5·51)
	Mixed model for repeated measures[b]				
	LS mean (SE)		0·6 (0·63)		0·3 (0·93)
	95% CI		(-0·7, 1·8)		(-1·5, 2·2)
	LS mean difference (OCS-01 – Vehicle) (SE)		0·2 (1·12)		
	95% CI		(-2·0, 2·4)		
	p-value		0·843		
AUC[d] of BCVA ETDRS letter changes across postbaseline visits up to Week 6 and Week 12 compared with baseline[a]					
Up to Week 6	AUC through Week 6				
	n	100		48	
	Mean (SD)	197·64 (256·332)		103·59 (237·660)	
	Median	181·00		108·50	
	Min, max	-442·5, 1008·5		-785·5, 640·5	
	Analysis of covariance[e]				
	LS mean (SE)	198·2 (25·08)		102·4 (36·21)	
	95% CI	(148·6, 247·8)		(30·9, 174·0)	
	LS mean difference (OCS-01 – Vehicle) (SE)		95·8 (44·06)		
	95% CI		(8·7, 182·8)		
	p-value		0·031		
Up to Week 12	AUC through Week 12				
	n	100		48	
	Mean (SD)	518·30 (612·763)		245·51 (527·141)	
	Median	453·25		188·25	
	Min, max	-790·5, 2244·0		-1128·5, 1494·5	
	Analysis of covariance[e]				
	LS mean (SE)	518·2 (58·88)		245·7 (85·01)	
	95% CI	(401·8, 634·6)		(77·7, 413·7)	
	LS mean difference (OCS-01 – Vehicle) (SE)		272·5 (103·45)		
	95% CI		(68·0, 477·0)		
	p-value		0·009		

Abbreviations: ANCOVA, analysis of covariance; AUC, area under the curve; BCVA, best corrected visual acuity; EOS, end of study; ETDRS, Early Treatment Diabetic Retinopathy Study; ITT, intent-to-treat; LS, least squares.

[a] Baseline was defined as the last non-missing measure prior to initiation of study drug. [b]Change from baseline compared between treatment groups was analyzed with a mixed model for repeated measures using an Autoregressive 1 covariance structure, patient as a random effect, fixed effects for treatment and visit, the interaction between treatment and visit, and baseline BCVA ETDRS letter score as a covariate. [c] Baseline was defined as the last non-missing measure prior to the first dose of the 3 times a day dosing regimen (ie, 6). [d]AUC was computed for change from baseline in BCVA ETDRS letter score through Week 6 or Week 12 using observed data and the trapezoidal rule. [e]AUC compared between treatment groups using an ANCOVA model with treatment and baseline BCVA ETDRS. Imputation rules were applied based on a pattern-mixture model approach. Table header N's indicate the number of patients contributing data to the statistical modeling and n indicates the number of patients with data available prior to imputation.

Table S5 Proportions of patients with a 15 letter/3 line or greater gain from baseline in ETDRS BCVA at each visit (ITT Set)

Visit		Vehicle (N=48)	OCS-01 (N=100)
Week 2	n	45	97
	>=3 Line Gain (response), n(%)	1 (2·2)	10 (10.3)
	<3 Line Gain, n(%)	44 (97·8)	87 (89.7)
	Estimated Marginal Effects of Treatment	2·26	10.15
	95% Confidence Interval	-2·11,6·62	4.19,16.12
	Difference in Marginal Effects (OCS-01 - Vehicle)	7·89	
	95% Confidence Interval	0·48,15·31	
	p-value	0·037	
	Adjusted Odds Ratio (OCS-01 / Vehicle)	5·00	
	95% Wald Confidence Interval for Odds Ratio	0·62,40·43	
	p-value	0·131	
Week 4	n	45	96
	>=3 Line Gain (response), n(%)	5 (11·1)	17 (17.7)
	<3 Line Gain, n(%)	40 (88·9)	79 (82.3)
	Estimated Marginal Effects of Treatment	10·57	17.35
	95% Confidence Interval	1·81,19·34	9.83,24.87
	Difference in Marginal Effects (OCS-01 - Vehicle)	6·77	
	95% Confidence Interval	-4·79,18·33	
	p-value	0·251	
	Adjusted Odds Ratio (OCS-01 / Vehicle)	1·78	
	95% Wald Confidence Interval for Odds Ratio	0·61,5·16	
	p-value	0·291	
Week 6	n	41	95
	>=3 Line Gain (response), n(%)	4 (9·8)	24 (25.3)
	<3 Line Gain, n(%)	37 (90·2)	71 (74.7)
	Estimated Marginal Effects of Treatment	9·40	24.40
	95% Confidence Interval	0·70,18·11	15.89,32.91
	Difference in Marginal Effects (OCS-01 - Vehicle)	15·00	
	95% Confidence Interval	2·80,27·21	
	p-value	0·016	
	Adjusted Odds Ratio (OCS-01 / Vehicle)	3·14	
	95% Wald Confidence Interval for Odds Ratio	1·02,9·70	
	p-value	0·047	
Week 8	n	39	93
	>=3 Line Gain (response), n(%)	5 (12·8)	24 (25.8)
	<3 Line Gain, n(%)	34 (87·2)	69 (74.2)
	Estimated Marginal Effects of Treatment	12·24	24.58
	95% Confidence Interval	2·37,22·12	16.07,33.10
	Difference in Marginal Effects (OCS-01 - Vehicle)	12·34	
	95% Confidence Interval	-0·68,25·36	
	p-value	0·063	
	Adjusted Odds Ratio (OCS-01 / Vehicle)	2·36	
	95% Wald Confidence Interval for Odds Ratio	0·84,6·61	
	p-value	0·103	
Week 12/EOS	n	40	84
	>=3 Line Gain (response), n(%)	3 (7·5)	23 (27.4)
	<3 Line Gain, n(%)	37 (92·5)	61 (72.6)
	Estimated Marginal Effects of Treatment	7·81	23.76
	95% Confidence Interval	-0·46,16·09	15.26,32.26
	Difference in Marginal Effects (OCS-01 - Vehicle)	15·95	
	95% Confidence Interval	4·00,27·90	
	p-value	0·009	
	Adjusted Odds Ratio (OCS-01 / Vehicle)	3·75	
	95% Wald Confidence Interval for Odds Ratio	1·07,13·23	
	p-value	0·039	

Note: EOS=End of Study. Baseline is defined as the last non-missing measure prior to initiation of study treatment. Model used is a generalized estimating equation for binary response model with fixed effects for treatment and visit, the interaction between treatment and visit, and baseline BCVA ETDRS letter score as a covariate. Estimated marginal effects, difference in estimated marginal effects, and confidence interval limits from analysis are multiplied by 100. Imputation rules are applied based on a pattern-mixture model approach; table header N's indicate the number of subjects contributing data to the statistical modelling.

Table S6 Changes from Baseline in CST (ITT Set)

		OCS-01 (N=100)		Vehicle (N=48)	
Visit	Statistics	Actual Value	Change	Actual Value	Change
Change in CST at Week 6 compared with baseline[a]					
Baseline	N	100		48	
	Mean (SD)	453·0 (131·81)		445·3 (112·46)	
Week 6	N	95	95	41	41
	Mean (SD)	388·5 (114·36)	-68·3 (111·53)	443·5 (110·96)	6·3 (77·84)
Mixed model for repeated measures[b]					
	LS mean (SE)		-63·6 (8·27)		5·5 (12·11)
	95% CI		(-79·8, -47·4)		(-18·2, 29·3)
LS mean difference (OCS-01 - Vehicle) (SE)			-69·1 (14·59)		
	95% CI		(-97·7, -40·5)		
	p-value		<0·0001		
Change in CST at Week 12 compared with baseline[a]					
Week 12/EOS	N	84	84	40	40
	Mean (SD)	369·4 (92·90)	-73·0 (107·41)	423·5 (90·07)	-12·8 (86·50)
Mixed model for repeated measures[b]					
	LS mean (SE)		-61·6 (9·13)		-16·0 (13·00)
	95% CI		(-79·5, -43·7)		(-41·5, 9·5)
LS mean difference (OCS-01 - Vehicle) (SE)			-45·6 (15·91)		
	95% CI		(-76·8, -14·4)		
	p-value		0·004		
Change in CST at Week 12 compared with Week 6[c]					
Week 6[c]	N	93		40	
	Mean (SD)	384·6 (110·85)		438·3 (107·22)	
Week 12/EOS	N	84	84	39	39
	Mean (SD)	369·4 (92·90)	-1·9 (59·93)	418·6 (85·65)	-18·7 (91·80)
Mixed model for repeated measures[b]					
	LS mean (SE)		-2·8 (8·30)		-11·6 (11·07)
	95% CI		(-19·1, 13·5)		(-33·3, 10·1)
LS mean difference (OCS-01 - Vehicle) (SE)			8·8 (14·01)		
	95% CI		(-18·7, 36·3)		
	p-value		0·530		

Abbreviations: CST, central subfield thickness; EOS, end of study; ITT, intent-to-treat; LS, least squares.
[a] Baseline was defined as the last non-missing measure prior to initiation of study drug.
[b] The change from baseline compared between treatment groups was analyzed with a mixed model for repeated measures using an Autoregressive 1 covariance structure, patient as a random effect, fixed effects for treatment and visit, the interaction between treatment and visit, and baseline CST as a covariate. Imputation rules were applied based on a pattern-mixture model approach. Table header N's indicate the number of patients contributing data to the statistical modeling and n indicates the number of patients with data available prior to imputation.
[c] Baseline was defined as the last non-missing measure prior to the first dose of the 3 times a day dosing regimen (.ie, Week 6).

Table S7 Non-ocular adverse events in 2 or more patients at by system organ class and preferred term (Safety Set)

System Organ Class<br>Preferred term	n (%) patients<br>OCS-01<br>N=100	 Vehicle<br>N=48	 Total<br>N=148
Any non-ocular TEAE	40 (40·0)	15 (31·3)	55 (37·2)
Infections and infestations	9 (9·0)	6 (12·5)	15 (10·1)
COVID-19	2 (2·0)	2 (4·2)	4 (2·7)
Nasopharyngitis	2 (2·0)	1 (2·1)	3 (2·0)
Bronchitis	1 (1·0)	1 (2·1)	2 (1·4)
Cellulitis	1 (1·0)	1 (2·1)	2 (1·4)
Vascular disorders	12 (12·0)	2 (4·2)	14 (9·5)
Hypertension	10 (10·0)	1 (2·1)	11 (7·4)
Metabolism and nutrition disorders	6 (6·0)	3 (6·3)	9 (6·1)
Diabetes mellitus	3 (3·0)	0	3 (2·0)
Type 2 diabetes mellitus	2 (2·0)	1 (2·1)	3 (2·0)
Nervous system disorders	7 (7·0)	2 (4·2)	9 (6·1)
Dizziness	3 (3·0)	0	3 (2·0)
Dysgeusia	3 (3·0)	0	3 (2·0)
General disorders and administration site conditions	5 (5·0)	2 (4·2)	7 (4·7)
Chest pain	1 (1·0)	1 (2·1)	2 (1·4)
Oedema peripheral	2 (2·0)	0	2 (1·4)
Investigations	4 (4·0)	2 (4·2)	6 (4·1)
Blood glucose increased	2 (2·0)	0	2 (1·4)
Gastrointestinal disorders	3 (3·0)	1 (2·1)	4 (2·7)
Constipation	1 (1·0)	1 (2·1)	2 (1·4)
Musculoskeletal and connective tissue disorders	3 (3·0)	0	3 (2·0)
Arthralgia	2 (2·0)	0	2 (1·4)

Abbreviations: PT, preferred term; SOC, system organ class; TEAE, treatment-emergent adverse event.

Figure S1 Change in BCVA ETDRS letter score from baseline to Week 12 (ITT Set)

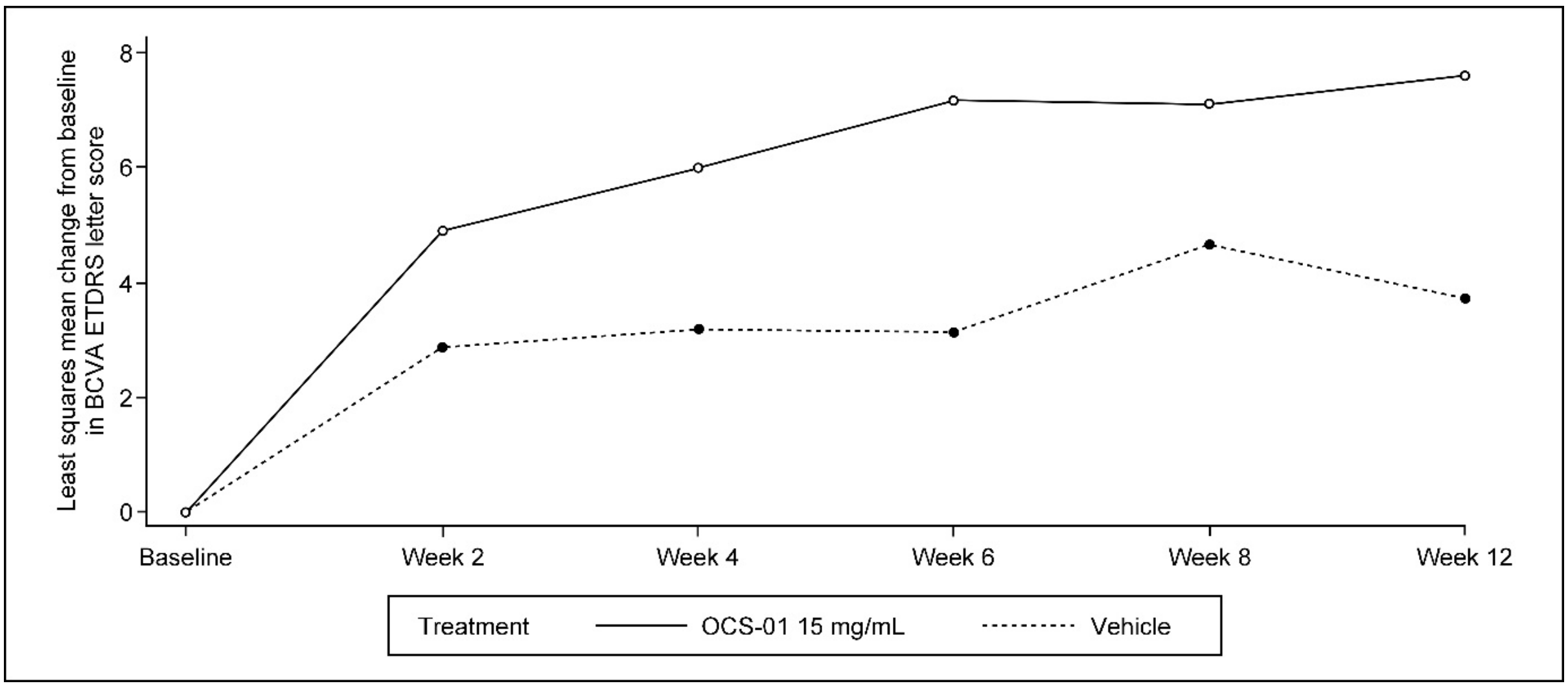

Abbreviations: BCVA, best corrected visual acuity; EOS, end of study, ETDRS, Early Treatmentb Diabetic Retinopathy Study; ITT, intent-to-treat

Note: baseline was defined as the last non-missing measure prior to initiation of study drug. The change from baseline compared between treatment groups was analyzed with a mixed model for repeated measures using an autoregressive 1 covariance structure, patient as a random effect, fixed effects for treatment and visit, the interaction between treatment and visit, and baseline BCVA ETDRS letter score as a covariate. Imputation rules were applied based on a pattern-mixture model approach
